# Vulnerability and Well-Being Decades After Leaving Care

**DOI:** 10.3389/fpsyg.2021.577450

**Published:** 2021-01-27

**Authors:** Thomas Gabriel, Samuel Keller, Clara Bombach

**Affiliations:** ^1^Zurich University of Applied Sciences, School of Social Work, Institute of Childhood, Youth and Family, Zurich, Switzerland; ^2^Marie Meierhofer Children’s Institute, Zurich, Switzerland

**Keywords:** well-being, vulnerability, long-term outcome, residential care, Child Care Research, life-course perspective

## Abstract

One of the most important goals of out of home placements is to reduce vulnerability and to enable well-being in the long term. This article hermeneutically reconstructs biographies decades after leaving-care to understand the impact of residential care experiences on selected dimensions of care-leavers’ well-being, that were discovered in the data material. For this article three analytic areas were selected from the core of the narratives of former care leavers: Social networks, parenthood and state interventions. The selected findings on long-term outcomes presented here are based on a qualitative research project funded by the Swiss National Science Foundation on life trajectories after residential care (1950–1990). The authors have conducted 37 biographical narrative interviews with former children placed in residential care between 1950 and 1990 in the Canton of Zurich, Switzerland. The analysis of these narrative interviews was structured by the inductive procedures of Grounded Theory. Its foundation is the conceptualisation and dimensionalisation of data through inductive coding within the narratives. Research question: We mainly were interested in aspects of transitions exclusively relevant from the actors’ point of view. The objective of this paper is to learn for the future by taking biographical experiences and long-term outcome in account. As we know residential care facilities have changed in last decades, but structurally some key figures are still continuing. They still interrupt the life course two times: when you start to the live in the institution and when you leave. One main question is how young people manage to integrate residential experiences through their life course and where they keep on struggling until the end of their lives. From a life-course perspective, the impact of social service intention on individual life courses, behind sending the individuals to such facilities, are important to investigate. They implicate relevant information concerning current practice and impact of placing children in residential care. Social networks and experiences of parenthood show why we must frame and accompany transitions out of care.

## Introduction

In Switzerland, over the past century, tens of thousands of children and young people have been placed in foster care and residential care. The same number only left these places many years later. The research shows that their well-being and the adolescents’ individual development were often of secondary importance after the placement went through, or after they left care (Lengwiler et al., 2013). Between 1950 and 1990, many child protection measures even culminated in penal institutions, and sometimes the adult penal system – a common administrative practice. It has been extensively confirmed that the children’s needs and the reasons for their behaviour played no role in placement decisions. More emphasis was placed on maintaining social order and conformity and the established power balance, entirely following the logic of those within the system who enjoyed power, authority and the right to act on behalf of the state.

In the highly federalised Swiss system, welfare, education and legal policy are largely a cantonal responsibility. National rules and regulations are imposed in specific cases only. Thus, this system does not rely on any developed federal bureaucracy or government agency. That is also why no federal Ministry of Child, Family or Welfare exists. Further, for both political actions and for professional family interventions conservative family ideologies still are implicit and often also explicit motivating forces in Switzerland. Low levels of interventions, but also less support and social security in an international comparison can be seen as consequences. The latest change of philosophies is marked by the new law on child and adult protection in 2012, which organises child protection in a more professional and coordinated way and gives more rights to children and families. Today, it is known that transitions in people’s lives are social phenomena which are not just subjectively experienced and embedded in everyday life but also a challenge for institutional regulations. They are always connected to conditions of uncertainty, unpredictability and possibility; to openness and contingency; to inequalities and differences; to chances of success and failure (Walther et al., 2019, p. 5). However, the few longitudinal studies to have been carried out in the field of children’s residential care show that when young people experience positive individual biographical developments on leaving residential care, this is often unexpected and usually goes against professionals’ predictions (Bullock et al., 1993). These findings are less an expression of individual resilience (Schofield et al., 2017) and more a sign of the poor understanding of the connections between institutions, biographies and society. The results of the study presented here show that the paths people’s lives take following residential care cannot be explained monocausally or following the logic of subsumption, based on individual, isolated risk factors they encounter as they grow up (neglect in the parental home or experience of stigmatisation; Gabriel and Keller, 2014). From a scientific point of view, it thus does not seem sufficient to consider individual influencing factors in isolation, as the various influences on well-being can be assumed to have an interactive dimension which is especially likely to surface during transitions and which can be reconstructed with some clarity.

## Materials and Methods

Transitions thus cannot simply be assumed to be a given; we need to expose and understand the practices which turn social processes into transitions. Thus, we carried out a biographical, reflective study involving people with experiences of residential care. Among other things, this was interested in how the interviewed actors made aspects of transitions relevant, and who was significant in this practice. We also wanted to know what social ties, structures and processes are expressed before, during and after residential care (Walther et al., p. 11), and how they are reproduced and have a relevant effect on people’s subsequent biographies. Our study takes into consideration journeys to adulthood out of residential care as a lifelong process for individuals based on their views, reconstructions and experiences narrated in biographical interviews (Rosenthal, 1993; Schütze, 2004).

As part of the Sinergia project ‘Placing Children in Care 1940–1990’, the subproject ‘Life trajectories after residential care placements in the canton of Zurich 1950–1990’ included biographical interviews with 37 former residents of children’s homes in the canton of Zurich. The distribution of interviewed men and women over the decades (1950–1990) was balanced. There were also no major gender-related differences in these decades in terms of placements – only the reasons were long dependent on gender (Businger and Ramsauer, 2019). The earliest that any of the interviewees left residential care was in 1951; the latest entry was in 1989. This means that at the time of the interviews the interviewees were between 25 and 85 years old. The reasons for entering the children’s home and age on entry varied. A frequent reason for leaving was to start vocational training at the age of 16 to 18.

Former residents of homes for children and young people in the canton of Zurich between 1950 and 1990 were made aware of the research project through notices in the press, online and on handouts. The interviews were carried out in various places selected by the interviewees themselves, such as cafés, rooms at the university or sometimes the interviewees’ homes. They lasted between 2 and 5 h. The interviewer and the interviewee drew up an agreement saying that the information would be treated as strictly confidential and had to be anonymised before being transcribed or used. The interviewees were also able to withdraw the information they had provided or delete some of their statements. The names of the people cited in the present text have been anonymised for reasons related to research ethics and the law on personal security and data protection. Except for our interest and listening, no further incentives were promised. Travel expenses were covered and as a symbolic thank you, we gave sweets worth about 25 Euros. Many were willing to participate because they want todays residential care to learn from the mistakes of the past and/or because they want to tell their story. For some, it was the first time they had talked about their experiences in care; they had not wanted to speak about it to their partners, children or friends, usually as they were afraid of painful questions and memories. Others had found that, when they ‘confessed’ to having being brought up in care, their experiences were downplayed (‘it can’t have been that bad’), they were not believed or they were even accused of being partly responsible for their stigma.

A highly intensive and detailed analysis of selective biographical trajectories (Gilligan, 2009; Zeller, 2014) is a promising way to methodologically deal with biographical complexities. In this context, it was of special interest to scrutinise the biographies, which would then make it possible to hermeneutically reconstruct (Rosenthal, 1993; Schütze, 2004), understand and analyse the biographies of adults with residential care experience. In research on transitions (Henderson et al., 2009; Sherif and Sherif, 2009), institutional transitions (school, profession, residential care, etc.), but also unforeseen biographical events are mostly focused on in their structural contexts. Reflective processing on the part of the subjects is of central importance here, as is their environment throughout their entire lifespan. According to this, narrated biographies are conditioned by subjective logics.

However, our biographical study’s research aims were not limited to any individual case, or to descriptively retelling subjects’ first-hand accounts of their life stories. Instead, the research was focused on grounding theories about intersubjective experiences of care leavers; contexts which came up repeatedly and gave structure to people’s overall or interim assessments of the lives they had lived, in relation to different biographical topics and dimensions of well-being. With their open narrative questions (Schütze, 2004), the interviews left plenty of leeway for non-directed memories and stories. Using the methods of Grounded Theory (Strauss and Corbin, 1990; Glaser, 2004), teams of analysts were able to extract central dimensions of well-being from the data by using the transcribed narratives. They reconstructed biographies and distinguished between them in an iterative process. All analytical steps were done in analysis-groups of three researchers to ensure their self-reflection.

This qualitative analysis of the narrative interviews was structured by the clear and extensive procedures and its straightforward approach to theory generation of Grounded Theory (Strauss and Corbin, 1990; Glaser, 2004). Its foundation is the conceptualization of data through inductive coding within the narratives – and further – data: the main three steps led us from open coding to axial coding and ended in selective coding which allowed us to inductively elaborate core variables; the analytical process was accompanied by constant comparisons and ‘memoing’ (Glaser, 2004). The product of this method is a set of grounded concepts. They are always integrated into inductively grounded hypotheses as well as organised around one core category. The goal is to explain the preponderance of behaviour in a substantive area: ‘The goal of grounded theory is to generate a conceptual theory that accounts for a pattern of behavior which is relevant and problematic for those involved. The goal is not voluminous description, nor clever verification’ (Glaser, 1978, p.93). As the character of this methodological access is not problem-centred but open, it was possible to inductively identify group-specific dimensions of experience in biographical transitions out of care and after care as well as within situations of daily living.

## Results

### Well-Being and Vulnerability in Selected Fields of Life and Its Connection to Experiences in Residential Care

Depending on how they are defined, sensitive and relevant transitions out of institutions or child and youth welfare measures cover various different time frames. One thing on which the discourse does agree on, however, is that transitions from residential youth welfare to adulthood take place in specific individual and structural conditions which have to be understood and included in social pedagogical endeavours (Stein, 2012; Köngeter et al., 2016; Refaeli et al., 2016). The effect of case-by-case, long-term support planning and intensive professional assistance after leaving the care structure would appear to be correspondingly strong.

Below, the biographical effects of experiences in care and during the wide range of transitions from care are addressed based on selected areas of life. These have emerged as key categories when it comes to the interplay between vulnerability and well-being in transitions and life courses after residential care. They also represent central nodes between the micro and meso levels and are related to each other: (1) social networks and social ties, (2) subjects’ own experiences of parenthood and (3) dealing with state interventions. Focusing on these fields of life, empirical material is used to shed light on the connections between subjects’ upbringing in and experience of residential care and the biographical topics. Although the selective examination of certain fields of life among subjects with experience of residential care calls for professional assessment, it should also be noted that, particularly in sensitive phases of people’s lives, topics come up which are related not only to the effects of residential care but also to their entire previous biography.

#### Social Networks and Social Ties

Subjects’ personal social network, seen as a resource for coping with life, influences the way they deal with critical life events centrally (Stein, 2008; Melkman, 2017). What is important in this context is the quality of the support and whether and how an individual can take advantage of support, in what situations. In the case of people who were taken into care as children, Freisler-Mühlemann (2011) shows that after leaving care, they render their social networks ‘unusable’ through their own behaviour. Others have social connections, but are revealed to find it challenging to know when and how to use them (Melkman, 2017).

Between 1950 and 1990, social ties were monitored and sanctioned in homes for children and young people. Carers reacted with suspicion to social connections, ‘deeper emotional relationships’ between children and adolescents, and the formation of groups (Hafner, 2014). Contact between children and adolescents was thus strictly controlled and minimised where possible. Everyday life in residential care was tightly structured through rules, discipline and order. Feelings of empathy and safety – the prerequisites for building trust between children in homes and their carers – were few and far between (Bombach et al., 2017). The top priority was on managing the children and young people as a group; individual needs came second.

In these conditions, children’s homes were rarely seen as safe places (Bettelheim, 1974) from the care leavers’ point of view. Attachment figures, who play a key guiding role in children’s socialisation, appear in their memories of everyday life in care home as taking advantage of their power, abusing, mistreating or neglecting children (Backes, 2012). Stanulla (2003) thus poses the question of whether, and how, they can regain trust in other people, and indeed in themselves, after leaving care, when that trust has become fragile or been lost altogether. The fact that this can become a central challenge and a lifelong task is clear from reports by former residents of children’s homes describing feelings of emotional distance from other people (Kuhlmann, 2008).

##### Loneliness and isolation within the group

Feelings of loneliness, isolation and being left to their own devices are present in the narratives. The feeling of being unwelcome or superfluous is clearly expressed in the following quotes: ‘*Yeah, sure. God, they could have just put an end to us instead*’ (*Jonas*). ‘*You were simply superfluous, like a chunk of meat, except we were still alive’* (*Jonas*). These dehumanised descriptions clearly illustrate how former placed children see themselves, as their lives at the home, as one of many, appear to be worth little.

The loneliness experienced within the numerically large group of children in residential care can be explained when the social matrix of the peer group is examined (Polsky, 1962; Gabriel, 2009). Outwardly, children in residential care appear to be a homogeneous group, in the 1960s partly because they looked the same, e.g., having the same clothes or hairstyles. Travelling together on their way to the external school, children from children’s home are described as a close group who stand up for one another and join together in solidarity when there is conflict with the ‘other, normal’ children. At the same time, the social structure of the children from the residential care follows its own, different rules (Emond, 2003). The need to assert themselves as individuals within the group is often described: ‘*The ones who don’t manage to battle their way through alone, let’s just say, go under’* (*Jonas*). In that context, the group offers no protection and can even exacerbate experiences of discrimination and exclusion. Many former placed children reported that they tried to get away from everyday life at the residential home – even if it was often only for short periods of time – for example by roaming through the woods alone, escaping supervision and enjoying the feeling of wandering about aimlessly. At the same time, social contacts were increasingly perceived as risky and avoided (Bombach et al., 2017).

##### Significant adults: powerful and rare

The children experience a very broad range of relationships with the carers during everyday life in residential care, offering an insight into the highly individual way in which children and young people are treated there. Affection or positive reinforcement are experiences which rarely come up in their memories: ‘*But otherwise, love or that, or trust in any one teacher or carer or that, forget it’* (Jonas). From the late 1960s there was an increasing tendency for interns who were still in training to work in residential care (Bombach et al., 2018b). They were described by former residents as people who are interested in the individual child. Generally, it can be said that, in stories by former placed children, ‘significant other’ (Courtney et al., 2001, p. 697) appeared who evidently had scope for action, which they put to use in highly individual ways and thus had a major impact on children’s journey through life. It can be seen that people acting in a child-centred manner in or outside the children’s home sometimes became important sources of power for children and young people. It is also striking that non-educational staff such as the cook, laundress or gardener took on the role of attachment figures who looked after the children with something like maternal or paternal care. As long-term attachment figures who were chosen by the children themselves, did not punish them and at the same time provided certain resources (e.g., food), they were important in helping them grow up (Bombach et al., 2018b).

##### Agency after leaving care: the wish to finally control their own lives

From the point of view of the former placed children we interviewed, on leaving care their aim was frequently to escape from the networks which they had not chosen freely while in residential care and were primarily linked to monitoring and sanctions: ‘*No-one could tell me what to do any more; I could live my own life and actually did a good job of it; I don’t owe anyone anything’* (*Jonas*). For many former residents, their own lives thus only began after they left the children’s home. Their relationship with themselves is then often described as the only relationship they can rely on. For some interviewees, the time they spent in residential care means that opening up to other people, being trusting and letting go of control becomes a task that is related to the responsibility of working on themselves as people: ‘*And that’s the problem if, as an adult, you simply know from experience that you can’t rely on anyone or anything and that if you don’t manage to do something yourself, no-one else will’* (*Alex*). Becoming attached to new places, things and people is often described as a challenge, with particular emphasis placed on their life as a loner. Meanwhile, as they had few experiences of community and relationship-building to draw upon, when it came to building and maintaining social relationships, constant attacks and defence were frequently a biographical topic.

##### Social and emotional scepticism towards others and themselves

Generally, it can be said that the people who were interviewed display great social and emotional scepticism. This distrust was expressed not only towards other people, but also towards themselves. Among other things, this is due to the experience of stigmatisation among former residents of children’s homes, which may persist throughout their lives, repeatedly exposing them to the experience of *I do not belong and am different* (Goffman, 1963). Being able to open up to relationships with friends, partners and children is often described as a major difficulty which affects almost all children in residential care. ‘*Very difficult, because you never really trust anyone. You don’t know the basic trust that a child enjoys’* (*Alex*). This often means that former placed children no longer run the risk of entering into relationships, or are not willing or able to get that close to other people. The interviews describe their distant ‘social coldness’ and lack of any expectations towards other people, including as a coping strategy that enables them to actively, and thus autonomously, counteract any disappointments and uncontrollable situations that might arise: ‘*People often say that there’s a certain hardness about my feelings, but, yes, that might be the case, but it just came about because of life somehow, […] the first ten or fifteen years. I’m not emotionally stunted; not at all, but at some point I put on the brakes a bit and don’t go any further’ (Franz).* In the following quote, it becomes even clearer that the fear of being hurt can block people to the extent that they have no social ties, and always have to do their coping alone: ‘*I can’t open up because I’m afraid of being hurt. That is, in all my life I’ve never had a friend I could simply trust. There’s no-one like that in my life, so I just go through it all on my own’* (*Nora*). This interviewee went on to report that: ‘*I don’t have any viable relationships outside my family and I think that’s a shame’ (Nora).* At the age of almost 50, after an accident she realised that she did not know anyone who could have bought groceries for her.

The challenge of creating social connections was most evident when seen in contrast: stable relationships were cited as proof that people had ‘made it’; succeeded in living with someone in a manner recognised by society. One point which stood out was that, of all types of connection, long-term relationships with partners or friends were possible when these had had similar experiences in their childhood.

The way subjects dealt with their own experience at the children’s home varied from going on the offensive to hiding their actual social identity, which extended, for example, as far as a strategy of deception: some interviewees reported that they did (or wanted to do) things that they did not think children in residential care were entitled to, such as driving a limousine, living in an upmarket neighbourhood of Zurich or owning a home. These findings are complementary to other study results, which underline the high relevance of often forgotten social aspects and networks in and throughout residential care (Stein, 2012; Melkman, 2017; Schofield et al., 2017; Ammann and Schwendener, 2019).

#### Experiences of Own Parenthood

Baader (2014) offers evidence of how strongly their childhood experiences affect former residents of children’s homes when they become parents, and of secondary traumatisation in the subsequent generation. According to Kuhlmann (2008), raising their own children becomes a challenge as it is related to their fears of repeating their own childhood experiences. Rosenthal (2010) describes similar intergenerational effects in another context. In her intergenerational studies on how people process their past under Nazism, she shows how they take on an identity as a victim and the following generation display signs of pseudo-identification. Dealing with that challenge can thus result, for example, in cold distancing, being overwhelmed by the children’s needs and a general attempt to hide their own experiences from the children. Ionowlock (1993) is another writer to point out that the next generation suffers more if the parents’ traumatising experiences are not discussed.

##### Disrespect, integrity and recognition

Together, the experience of many former placed children showed that they were denied central dimensions of recognition during their childhood. Alongside experiences of physical violence, Honneth (1992) describes experiences of disrespect as being of far-reaching effect. This type of experience relates, for example, to family interactions which infringe in a non-violent manner on people’s needs and entitlement to affection, respect and appreciation – that is, their needs and entitlement to recognition. Experiences of disrespect can harm their trust in themselves and the world, impacting not on their physical integrity but on their mental and social integrity. When examining the mutual recognition between generations, the term ‘*reconnaissance’* (Ricoeur, 2006), as used in social philosophy, seems fruitful. This has both an active and a passive dimension:

• active (‘*reconnaître*’): recognising something; things, people, someone else, one another
• passive [‘*(demander à) être reconnu*’]: being recognised, asking/demanding to be recognised.

Recognition thus moves away from the act of ‘mere *connaissance*’ (in the sense of the mastery of meaning) and, thanks to Ricoeur’s addition of the passive expectation of ‘wanting to be recognised’, becomes a dialogical form of recognition that can be satisfied only by mutual recognition (Ricoeur, 2006). This dialogical, interactive component of ‘*reconnaissance*’ as the basis of abilities, acquired through socialisation, to recognise oneself and others, forms the link to the findings on intergenerationality discussed here in the context of people’s own biographical experiences. The experience of not being ‘recognised’ by their parents often plays a central role in the biographies of former children’s home residents. Among people who were placed in residential care in early childhood, the question of the legitimacy or illegitimacy of their own birth is notably of lifelong significance. In the following quote, for example, the intergenerational links between recognition and disrespect becomes clear: ‘*I placed my trust in my mother, but she went off to Spain. I can’t rely on my father; he said that a friend of his was also there during group sex, and you came out of it by accident, and that was my father; he told me that too at 18, and then I knew I didn’t have a father’ (Paul).*

##### Violation of integrity

Both nationally and internationally, studies indicate that the mortality rate is higher among people who have experienced residential care. A now somewhat dated study by Tanner showed a mortality rate throughout Switzerland of 10% (9.3% in French-speaking and 11.3% in German-speaking areas; Tanner, 1999). Suicide and life-threatening risky behaviour can be understood as a radical answer to the central, basic question on integrity posed by Pollmann: ‘Is my own life worth living?’ (Pollmann, 2005). If the answer is negative or ambiguous, this can be a sign of fundamental disruptions to their integrity, or even its total loss. ‘Fear’ and ‘depersonalisation’ are emotional indicators that a person’s integrity may have been disrupted. According to Pollmann’s definition, people have integrity if, in a manner relatively free from internal and external constraints, they are able to live life

– (a) in accordance with their own, firm will,
– (b) within the limits of the morally tolerable, and
– (c) on the basis of an integrated ethical and existential self-understanding, and
– (d) with a general feeling of wholeness, which at the very least requires them to be mentally and physically unscathed (Pollmann, 2005).

Many reports of experiences in residential care describe ‘invasive encroachments’ on the integrity of children and young people in care by peers and adults (Bombach et al., 2018d). Feelings of ‘shame’ and ‘guilt’ for being placed in a home are particular indicators that their integrity has been violated, most clearly among people who have kept their experiences in the children’s home a secret from their children and partners to the present day. One aspect which seems to be central in this regard is the social dimension of integrity in the context of reappraising and publicly addressing the history of residential care. A lack of understanding (‘Many people were even beaten in their own families in the 1950s’) or a failure to recognise experiences in care which damaged their integrity can cause suffering and further undermine the integrity of the people affected (Pollmann, 2005). If, however, former residents of children’s homes were able to see and understand the reason why they were placed in care in the situation and conditions of the time, the issue of guilt was less likely to arise, and their integrity, and/or that of other people involved, was far less badly impaired, including the effects this had on the ways they saw themselves in the present day.

##### Tabooed sexuality in residential care

The descriptions of everyday life in residential care show that there were not many ways for children to locate self or become empowered, as the focus was more on managing large groups than on the children’s individual needs (Bombach et al., 2017). They became physically and psychologically accustomed to strict schedules. There was hardly any privacy; children shared bedrooms and bathrooms divided by sex. Sexuality, getting to know their own body or coming into contact with the opposite sex were taboo subjects. The young people rarely had relationships, and those who did had to do so in secret, as it was rarely tolerated by the homes’ managers. The same was true of relationships between the young people and staff at the children’s home. No lessons were planned on perceptions of their own or the opposite sex, on how to deal with intimacy and physicality, how to work on relationships, get the right balance between distance and closeness, or how to view themselves or others – and more than that, these subjects were actually suppressed: ‘*They didn’t teach us how to deal with the opposite sex. They always kept us apart. We didn’t have any contact with one other’ (Marie).* In these conditions, it was almost impossible for them to learn how to deal with their own needs, their body and their own sexuality. For many former residents, self-care remained a challenge long after they had left residential care. Their first sexual experiences were often described as highly ambivalent: ‘*I didn’t know what to do! And I was defenceless, too. I mean, if someone wanted something, I couldn’t say no even if I wanted to say no’ (Marie).*

##### Becoming a parent – a confrontation with their own childhood experiences

Having children or starting a family was a topic addressed in all the biographical interviews. Each subject described how, before their first child, they had engaged with the subject in an ambivalent manner, adopting a cautiously reticent or strongly negative attitude always based on their experiences in their own childhood. The following quote is representative of the greater number of men than women who decided against having children, sometimes at an early age – in this case on leaving care at age 16: ‘*There’ll never be any children for me, because if someone takes the children away I’ll run amok. And that’s why it’s always been a taboo for me, and I’ve never married, I’ll never have children, nothing. I didn’t want that any more because I’m afraid of that sort of thing, that really affected me’ (Jonas).* In this case, this firm decision represents protective empowerment, focusing on his own wishes and ensuring that these formative experiences cannot be repeated.

One woman who was interviewed saw herself as being forced to deal with her own past due to her partner’s wish to have a child. This triggered feelings of inability and insecurity; she distrusted her skills as a mother, which also offered an insight into her self-image: ‘*I always thought that I couldn’t have children with my past; that it wouldn’t be good; that I couldn’t be a good mother for the children. I didn’t feel capable’ (Marie)*. When her daughter was born, she swore that she would never let her down, and that she would always be there for her. However, she was not able to keep that promise. She described her inability to take action on recognising her daughter’s needs: ‘*And I did notice it at the time, but I couldn’t change it’* (Marie). The reservations she described before she became pregnant were confirmed in a self-fulfilling prophecy (Merton, 1948): ‘*I have the feeling that I made a lot of mistakes and she definitely needed something totally different and didn’t get it […]. And now, in retrospect, I’m simply sad that I didn’t do right by her’ (Marie).*

For another woman, encountering her own children brought memories of her own experiences in residential care back to the surface, confronting her with aspects of the past that she had, as she said herself, ‘consigned to a dusty shelf’. The long-suppressed or long-forgotten past suddenly and inadvertently sprang back to life, triggering several personal crises. Watching her children play, she realised: ‘*Playing, being happy. As my children got older, I went through a lot of personal crises as I saw what I’d missed myself: I wasn’t able to be a child’ (Nora).* Grieving for her own childhood experiences, she also underwent an uncontrollable freeze response. When it came to riding a bike, for example, her body refused point-blank to cooperate: ‘My husband once said, when the children were smaller, “Come on, darling, try it again now”. […] No, it doesn’t work, it doesn’t work at all. I wanted to do it though, for the sake of the children.’ *(Nora).* As a consequence, she set her own boundary of never trying cycling again, which brought her from a powerless position to a more powerful one. While she was forced to ride a bicycle during everyday life at the home, as an adult woman she had the choice, and was able to decide not to do so.

Other former children’s home residents also cautiously re-interpreted the damaging hardships of their childhood in a positive light: ‘*Yeas, sometimes I’m glad that I didn’t grow up in a family. When I think about all the things I’ve seen in families, sometimes I’m almost glad. I had to invent myself’ (Robert).*

#### Dealing With State Interventions

Between 1950 and 1990, many child protection measures (sometimes also ‘compulsory measures’) ultimately led to criminal justice institutions, and sometimes even adult prisons (Lengwiler et al., 2013). This was the case for some of the women interviewed: after running away from the children’s home, they were tracked down by the police, then always placed in even more secure accommodation before finally, in the following case, ending up as a minor in a women’s prison: ‘*I was away for about 3 months, and then when I came back I had to go to the women’s prison and then I never went back to youth custody. And then I came in with the murderers and criminals. At 15 or 16; yes, I was 15, not yet 16’ (Hedi).* Justifications on the part of the authorities did not relate to any explicit violation of the law, but instead to a lack of discipline and failure to adapt to the residential care setting, or to the risk of them running away again. To this day, secure accommodation supports the logic of the criminal law relating to young offenders and the youth welfare system, but this is strongly criticised (Peters, 2014).

Whatever the reason why children are placed in care, and wherever they are placed, when they have no contact with the justice system (if there is proof they have not re-offended) during and especially after residential care, this is often seen by experts as a verifiable factor testifying to the success of residential care (Gabriel and Stohler, 2008; Stein, 2012; Gabriel and Keller, 2015). This type of evidence is a very popular basis for social policy decisions, but says very little about the motives and conditions which eventually led to an act being reported as a violation of the law. Explanations based on the idea that patterns of delinquency are learned from other children and young people in the home (Bandura, 1997) or that the combination of a low level of schooling and poor families encourages them to achieve their social goals by illegitimate means [in the sense of anomie theory (Merton, 1938)] are to some extent confirmed by the interviewees’ memories of growing up in residential care: ‘*And that’s how you learned everything, firearms and drugs, breaking into cars, simply everything. […] After 2 years in the children’s home I was so well trained, I had no respect for anything’ (Alex).* However, with their strong focus on the delinquent acts themselves, these explanations barely pick up at all on the related subjective meaning; the underlying explanation. As a salient addition to the perspective on possible motives for delinquent acts, it also becomes clear from the narratives that coming into contact with the justice system, or with other (directive) state interventions in general (social assistance, the tax authorities) later in life represents another experience of restricted autonomy and agency, and can be seen as another example of being dealt with in an inhuman manner (Bombach et al., 2018c).

##### Immunity to interventions, rules and punishments

Every time young people are placed in residential care – they themselves also use the prison-related phrases ‘*locked up’* or ‘*committed’* – this is a state intervention in their lives. The ‘inmates’ – as they repeatedly called themselves in the interviews – had no influence on this influential decision. This led to profound and far-reaching consequences regarding the conditions in which they later grew up, and is one of the reasons why the children mostly perceived the adults they came into contact with during their placement as abstract representatives of the authorities, and of a state that controlled them somehow, from somewhere. Because the responsibilities, justifications and objectives behind the process of their being taken into care almost always remained unclear from the point of view of the children and young people, they frequently developed a high degree of scepticism towards other people and above all towards civil servants and everything related to the state, which often lasted long after they had left care.

It was not just that the children and young people were helplessly at the mercy of state decisions and actions; they often also (consciously or unconsciously) learned how to deal with disciplinary mechanisms – in full accord with Erving Goffman’s ‘secondary adjustments’ Goffman (1961) – or how to deal with the culture of the other children and young people at the children’s home (Polsky, 1962). While children with few resources had to give in to the power mechanisms of formal or informal everyday life at the home, others tried to repurpose them to their own advantage, or escape them. As it was not directly possible to escape punishment (including physical punishment), almost all the children and young people increasingly developed what they described in retrospect as an ‘inner immunity’ to interventions, rules and punishment: ‘*Like many children, I wasn’t actually bothered by punishment. My father had already dished it out a bit, so actually punishment didn’t really mean anything to me; nothing special, no, and it doesn’t hurt me either. That doesn’t mean that, in your head maybe either, but physically it doesn’t matter to me anyway’ (Michael).*

The young people almost always saw it as highly paradoxical that, for such a long time, they were not allowed to leave care without official permission, only to be suddenly forced to leave at the end of the intervention. These contradictions between the worlds in and outside the home, as they saw and described it, were exacerbated by the lack of support, assistance and financial, social and spatial (accommodation) resources (Bombach et al., 2018a). As a result, after leaving residential care, as one interviewee put it, they had to try to ‘*integrate themselves’* (Alex; Bombach et al., 2018d). The temptation was correspondingly high to do so by making use of whatever opportunities arose, even if these were often contrary to the normative idea of social integration: ‘*Then I just thought, “Fine, then I’ll make sure I get my stuff myself” and then you just start stealing and at some point […] you start dealing’ (Karin).* In this context, almost all the interviewees also speak of other former residents who were or still are in prison having left care, or who have died from drugs and alcohol.

##### The state as a constant, omniscient and unjust opponent

As a result of these experiences, many former residents of children’s homes are today still quick to feel personally humiliated, repressed, under attack and monitored in all possible kinds of interactions with representatives of the state, without having any rationally defined reasons to feel that way: ‘*Anyone that wants to tell me what to do, the authorities, police, anything that has anything to do with that, I have a massive problem with them’ (Karl).* This is even more painful if, for example, as in the following quotes, contact with an office underlines or consolidates their precarity and continued dependence, even in their current situation in life*: ‘I’ve sometimes been treated like total dirt there. You’re simply put in a group and, above all, the social welfare office has access to your files! They start out by looking in those – What do we already know about this person? – and that’s how you’re judged’ (Heinz).* The experience of the woman quoted below is representative of many ex-residents: ‘*It’s the authorities that make me sick. I’m caught up in it again, the social welfare office, feel like […] back in prison. That is, you have to work there, when they have a place for you to work, if you don’t there are sanctions […]. They decide where I have to work and […] how much I have to work, not allowed to have a car, not meant to have a dog’ (Hedi).*

In many cases, the feeling of never having escaped the clutches of dependency, rules and monitoring since childhood leads to anger: ‘*The thought already came into my mind that they should chuck a bomb under the social welfare office’ (Karl)*, or helpless resignation: ‘*The state’s won’ (Alex).* This can go so far that big, omniscient systems are seen as lifelong opponents, in a kind of conspiracy theory. Every time, for example, they come into further contact with the judiciary, this is seen as proof that, even decades after leaving residential care, they are not accepted and are being unjustly punished: ‘*Then they took my driving licence away for 6 years because I crashed my car. But of course they knew my story, and so, of course I’ve always thought that if things were shit at home, or you’ve been in a children’s home or whatever, that’s a bad thing; it puts you in a bad light. […] Yes, […] of course you’re not worth as much […] as an illegitimate child from residential care’ (Paul).*

The basic consequence that they lose confidence in both themselves and the state or welfare state seems to have even more far-reaching effects: ‘*I have to respect the state’s laws, but the state doesn’t have to show me any respect, they can do anything’ (Alex)*. Although Alex feels regulated by the state, he is deprived of any entitlement to rely on being recognised by the state – which also deprives him of his status as a citizen. However, not wanting to be controlled by others is not the same as wanting anarchy. This extreme rejection of external directives can also lead people to incorporate their own self-monitoring: ‘*I’ve always had the feeling that I have to clean first, to clear up first, to tidy everything up first, before I am allowed to have any time off’ (Monika).* It seems clear that, as long as the acts of the state and welfare state are perceived as disempowering acts of humiliation, then during critical life events, former children’s home residents will remain unable to accept offers of support which would enable things to change. Instead, such acts appear to reinforce their position as outsiders or victims. In the case of punitive interventions by the judicial system – from fines to court hearings or imprisonment – they have an even stronger impression of that position and their biographically established experiences of helplessness and anger being reinforced (Bombach et al., 2018d).

## Discussion

The relevant, empirically established links between experiences in residential care and the biographical topics central to people’s well-being, as discussed here, clearly reveal how children’s experiences of being placed in care between 1950 and 1990 can manifest later in life. This finding can also be linked to other studies with a life course perspective (Brady and Gilligan, 2018; Gradaílle et al., 2018). This, in turn, underlines the current need to search for and find answers to critical questions about children’s residential care which do not involve putting an end to homes (as is frequently demanded), but are instead about creating ‘evidence-based’ expertise and quality criteria relating to children being in and leaving residential care (Holden et al., 2010). The present insights into the results of our study imply that there is significant potential for development on the level of individual cases, and at the same time rebut any assumptions of determination caused by socio-structural risk factors or people’s biological or genetic dispositions. As well as a re-assessment of how their upbringing, education and social circumstances affect their life course; however, it also brings to mind topics relating to institutional criticism and reform, such as relevance of participation and relationships, or necessary reflections on family metaphors, inconsistencies and contradictions (Kendrick, 2013; ten Brummelaar et al., 2018; Hauss, 2020). Thus, the quality of their social networks and the recognition and the subjective degree of free rein children enjoy inside and outside the children’s home appear to have an important influence on their biographical trajectories and various spheres of their adult life.

### How a Lack of Scope for Action in Care Defines Later Scope for Action

A reconstructive analyses of the biographies of former residents of children’s homes showed that explicitly restricting their scope for decision-making and action while they were at the home, in relation to their social integration, can be seen as a direct consequence of the logic behind their upbringing and disciplining at that time. The goal of adjusting their behaviour and appropriately integrating people who grew up in youth welfare into society hampered their access to education and stopped them from developing social networks and building up confidence in themselves and others in the long term. Other ways in which their scope for action was limited were not experienced directly in everyday life at children’s home, such as later parenthood or how they dealt with state interventions, but instead made themselves all the more noticeable in later encounters and opportunities, when past experiences were, so to speak, reproduced in current ones. In terms of the consequences discussed, both developments led, in particular, to long-term violations of their physical or mental integrity which they sometimes experienced, and indeed continue to experience, as severe. These then trigger a feeling of helplessness, shame and guilt, which can lead on one hand to resignation and social withdrawal, grief, anger and aggression or on the other to a pronounced sense of justice, to empathy or to resilience and ambition. Ultimately, these experiences show that being removed from their families and placed in care did not initially create any opportunities for them to emancipate themselves socially and/or socio-economically. Instead, their intersubjective experiences indicate that the stigma of being brought up in residential care causes their exclusion from education and social participation in life to be constantly reproduced – usually by others, but also, subsequently, by the subjects. Paradoxically, the professionals’ goals, such as enabling them to manage their own well-being, to become socially and economically independent, to develop self-esteem or participate in society and politics, are in fact made impossible, sometimes in the long term, by the children’s experiences of the (actual) professional interventions. At the same time, however, it can be seen that these episodes also enable them to experience empowerment when, after leaving care, they create and use new opportunities for action in an active, self-empowered manner which contrasts with their experience at the children’s home. Despite, or even because of the unfavourable conditions of their development, many succeed in proactively, independently shaping their own biographies; in the words of Werner and Smith: ‘Not all development is determined by what happens early in life’ (Werner and Smith, 1982, p.98).

### Individual Pressure to Prove Themselves Remains as Recognition Is Denied

Precisely because they have always experienced their own biographical scope for action being restricted and blocked, many former children’s home residents feel considerable pressure to prove themselves as adults. During the transitions into adulthood, the obligations of their disciplinary upbringing often turn into implicit obligations to make their own success or suffering visible, and thus proving it to everyone. Success, for example, is objectified by presenting status symbols, claiming superiority over the situation of other children in residential care or other minorities such as foreigners, or by seeing their own standards as the most important and questioning all other standards. Suffering, meanwhile, is made visible and brought up again and again by writing books, making television appearances on the topic or discussing it in networks for former placed children, or in the form of becoming politically active regarding inquiries and demands for compensation. Many, however, have chosen to place their past under a taboo among their close social relationships and consequently avoid making any connections to their experience in care when they experience either success or injustice – or at least not openly.

The way in which they prove what they have achieved, and prove themselves – constantly making comparisons and looking for recognition – also makes them vulnerable: they rarely receive the recognition they desire from other people or the state in a manner they would consider appropriate. However, it can lead to them becoming dependent on the opinions and assessments of other people who wield a certain discursive power or legitimising influence, such as lawyers, doctors, scientists or politicians. However, this does not contradict the experience described by many former children’s home residents, of being solely responsible for themselves and not being able to rely on anyone. This latter experience relates to their having to ‘fight their way through’ life, while the former aspect is linked to ‘making the struggle visible’. These different dimensions of dependency clearly remind science and research of their ambivalent role, caught between empowering and disempowering former residents of children’s homes, and thus of the extreme sensitivity required from them in this field.

For many of the children and young people, growing up in care was associated with the experience of isolation and a lack of care. While they were in the children’s home, one fact which frequently negated their personality, specific needs and individual experiences was being described as ‘*the children from the home’* or ‘*from the orphanage’*. This was, and still is, associated with various multifaceted attributions, simplifications and devaluations. For the former residents, being called the ‘*children from the home’* today still frequently means being of little interest as individuals with their own needs, views and individual behaviours. For some people who have experienced life at a children’s home, the label of ‘*children from the home’* follows them their entire life, and they often even internalise some of the attributions themselves. Paradoxically, the experience of professional intervention itself has in some cases made it impossible, even in the long term, to achieve professionally desirable goals such as an independent assurance of well-being, social and economic independence, self-esteem or even participation in social and political life. Future qualitative and quantitative research in these fields should focus on understanding how residential care could take recent as well as long-term needs and wishes of young people into consideration. More research questions are needed to find out how children in care can be empowered as individuals and in groups within an institutional framework: How and from which relevant persons can they learn to shape their lives in a self-determined way? How can we prevent them from feeling like ‘children in care’ all their lives? We need a better understanding of biographical turning points. Because turning points can show important interrelations between interventions and vulnerability in life-terms. But these different dimensions of dependencies clearly point out the ambivalent role of science and research between empowerment and disempowerment of former placed children and thus their necessary high sensitivity in this field.

## Data Availability Statement

The datasets presented in this article are not readily available for reasons of the specific importance data protection and research ethics in this sensitive field, no data sharing will be possible. For ethical research reasons, because biographical interviews still allow conclusions to be drawn about the person even with intensive anonymisation due to the still apparent life story. The fact that no data can be shared open source with the community is an ethical statement of principle resulting from the nature of the data in a highly sensitive, tabooed and not yet processed field with vulnerable groups. In short: biographical interviews cannot be anonymised by changing names and years. If you change much more, data become arbitrarily and thus cannot be analysed anymore. Due to the highly sensitive nature of the field, it is particularly important that anonymised interview data is only passed on for research purposes with the explicit consent of the interviewees. No request possible (see above).

## Ethics Statement

Ethical review and approval was not required for the study on human participants in accordance with the local legislation and institutional requirements. The patients/participants provided their written informed consent to participate in this study.

## Author Contributions

TG: head of the study, guarantor of integrity of entire study, study concepts, study design, acquisition, data analysis/interpretation, and manuscript final version approval. SK and CB: study design, literature research, field and data access, interviewing, data analysis/interpretation, manuscript preparation, manuscript editing, manuscript revision, and manuscript final version approval. All authors contributed to the article and approved the submitted version.

## Conflict of Interest

The authors declare that the research was conducted in the absence of any commercial or financial relationships that could be construed as a potential conflict of interest.
